# Development and validation of an automated Tomotherapy planning method for cervical cancer

**DOI:** 10.1186/s13014-024-02482-x

**Published:** 2024-07-08

**Authors:** Feiru Han, Yi Xue, Sheng Huang, Tong Lu, Yining Yang, Yuanjie Cao, Jie Chen, Hailing Hou, Yao Sun, Wei Wang, Zhiyong Yuan, Zhen Tao, Shengpeng Jiang

**Affiliations:** 1 Department of Radiation Oncology, Tianjin Medical University Cancer Institute and Hospital; National Clinical Research Center for Cancer; Tianjin’s Clinical Research Center for Cancer; Key Laboratory of Cancer Prevention and Therapy, Tianjin, China; 2 Department of Oncology, Tongji Hospital, Tongji Medical College, Huazhong University of Science and Technology, Wuhan, Hubei China; 3 Department of Radiation Oncology, Tianjin First Central Hospital, Tianjin, China

**Keywords:** Automated radiotherapy planning, Tomotherapy, Volumetric modulated arc therapy (VMAT), Cervical cancer

## Abstract

**Purpose:**

This study aimed to develop an automated Tomotherapy (TOMO) planning method for cervical cancer treatment, and to validate its feasibility and effectiveness.

**Materials and methods:**

The study enrolled 30 cervical cancer patients treated with TOMO at our center. Utilizing scripting and Python environment within the RayStation (RaySearch Labs, Sweden) treatment planning system (TPS), we developed automated planning methods for TOMO and volumetric modulated arc therapy (VMAT) techniques. The clinical manual TOMO (M-TOMO) plans for the 30 patients were re-optimized using automated planning scripts for both TOMO and VMAT, creating automated TOMO (A-TOMO) and automated VMAT (A-VMAT) plans. We compared A-TOMO with M-TOMO and A-VMAT plans. The primary evaluated relevant dosimetric parameters and treatment plan efficiency were assessed using the two-sided Wilcoxon signed-rank test for statistical analysis, with a *P*-value < 0.05 indicating statistical significance.

**Results:**

A-TOMO plans maintained similar target dose uniformity compared to M-TOMO plans, with improvements in target conformity and faster dose drop-off outside the target, and demonstrated significant statistical differences (*P*^+^ < 0.01). A-TOMO plans also significantly outperformed M-TOMO plans in reducing V_50Gy_, V_40Gy_ and D_mean_ for the bladder and rectum, as well as D_mean_ for the bowel bag, femoral heads, and kidneys (all *P*^+^ < 0.05). Additionally, A-TOMO plans demonstrated better consistency in plan quality. Furthermore, the quality of A-TOMO plans was comparable to or superior than A-VMAT plans. In terms of efficiency, A-TOMO significantly reduced the time required for treatment planning to approximately 20 min.

**Conclusion:**

We have successfully developed an A-TOMO planning method for cervical cancer. Compared to M-TOMO plans, A-TOMO plans improved target conformity and reduced radiation dose to OARs. Additionally, the quality of A-TOMO plans was on par with or surpasses that of A-VMAT plans. The A-TOMO planning method significantly improved the efficiency of treatment planning.

## Introduction

Cervical cancer ranks as the fourth most prevalent malignant tumor among women globally [1]. Radiotherapy has served as a critical treatment modality for patients with locally advanced, lymph node-positive, and/or high-risk cervical cancer [2–5], as well as the standard regimen for postoperative adjuvant therapy [6, 7]. Radiotherapy for cervical cancer could lead to severe toxic reactions in the gastrointestinal, urinary, and hematological systems [8, 9]. Optimizing dose distribution with advanced radiotherapy techniques could help to minimize the side effects [10]. Tomotherapy (TOMO) and volumetric modulated arc therapy (VMAT) are two widely used and effective external beam radiotherapy techniques for treating cervical cancer [11, 12]. In clinical practice, radiotherapy dosimetrists manually created treatment plans using treatment planning systems (TPS), which often require optimization with multiple iterations and trial-and-error to meet dose constraints. However, due to the large treatment area required for cervical cancer, a delicate balance between achieving sufficient tumor coverage and protecting organs at risk (OARs) is necessary. Furthermore, the variations in patients' anatomy make the process of manual planning complex, cumbersome, and time-consuming [13, 14]. The variations of planner's expertise, execution standards, and effort expended, potentially lead to inconsistent plan quality, which not only increase the risk of toxicity to OARs but also complicate the outcomes and interpretations of clinical trials [15]. Therefore, it is essential to enhance the plan quality and consistency.

The application of automated radiotherapy planning allows for procedures to be executed with as little human intervention as possible, effectively improving the quality, efficiency, and consistency of radiotherapy planning [16]. There are primarily two types of strategies for automated planning: strategies based on atlas prediction of optimization objectives and script-based strategies that emulate manual optimization. The atlas-based methods use data from previous radiotherapy plans to train models that predict dose distribution for new patients [13]. However, this method is highly dependent on the training datasets [17], and tends to struggle when adapting to new cases that differ significantly from the training data. In contrast, the script-based methods don’t require a prior training or learning step [18, 19]. In the radiotherapy of cervical cancer, particularly with the VMAT technique, mature automated planning methods have already been developed and have been proven to achieve better plan quality and greater efficiency than manual planning [20–23]. However, research into the automated planning for TOMO plans in cervical cancer remains unreported, suggesting room for improvement and development.

It is well known that the Precision (Accuray Inc., Sunnyvale, CA, USA) TPS for TOMO lacks the function for implementing scripts, whereas RayStation (RaySearch Labs, Sweden) TPS possesses functions for TOMO planning and scripting. In this study, we developed a script-based automated TOMO (A-TOMO) planning method for external beam radiotherapy in cervical cancer using RayStation TPS, and the A-TOMO plans were compared with those from manual TOMO (M-TOMO) planning and automated VMAT (A-VMAT) planning to validate its feasibility and effectiveness.

## Materials and methods

### Patients

In this study, we consecutively selected 30 cervical cancer patients who underwent TOMO treatment at our center in 2023. This study strictly adhered to the ethical principles outlined in the Declaration of Helsinki and the protocol was approved by the institutional review board and ethics committee at Tianjin Medical University Cancer Institute & Hospital. All participants provided written informed consent. Among these 30 patients, 26 patients underwent definitive radiotherapy, and 4 received postoperative radiotherapy. All the TOMO plans were manually designed, with all patients receiving a dose of 50.4 Gy in 28 fractions. The patients were treated in two positions: 12 in the prone position and 18 in the supine position. The average volume of the planning target volume (PTV) was 1347.45 ± 179.28 cc, ranging from 1122.82 to 1862.99 cc. The average overlapping rate of the bladder in the PTV was 37 ± 11%, with a range from 13 to 64%. For the rectum, the average overlap rate with the PTV was 52 ± 13%, ranging from 24 to 72%. The average overlapping volume of the bowel bag in the PTV was 115.79 ± 52.09 cc, ranging from 8.05 to 205.67 cc.

### CT simulation and Contouring

CT scans were performed to obtain images with a thickness of 5 mm. To ensure bladder filling, patients were instructed to drink 500 ml of water 30 min before the scans. Following the Radiotherapy Oncology Group (RTOG) guidelines, radiotherapy oncologists delineated the targets and OARs on the CT images, including the clinical target volume (CTV), PTV, bladder, rectum, bowel bag, femoral heads, and kidneys. For patients undergoing definitive radiotherapy, the CTV encompassed the uterus and its lymphatic drainage area. For those receiving postoperative radiotherapy, the CTV included the lymphatic drainage area of the radical uterus. The PTV was defined by expanding the CTV by 7 mm in all three dimensions.

### Radiotherapy planning

#### Radiotherapy planning protocol

The radiotherapy planning protocol in our center adhered to the dose limits for the PTV and OARs as recommended by the National Comprehensive Cancer Network (NCCN) guidelines and the recommendations of Quantitative Analysis of Normal Tissue Effects in the Clinic (QUANTEC), as detailed in Table 1. Here, D_max_ denoted the maximum dose, V_xGy_ represented the volume receiving x Gy in the dose volume histogram (DVH), and D_mean_ indicated the mean dose. This protocol of dose volume limits ensured the priority fulfillment of hard constraints for indexes, which meant that in case of conflicts between OARs protection and PTV coverage, partial prescription coverage of PTV could be compromised.

**Table 1:** Target and OAR dose constraints

ROI	Index	Constraint	Type
PTV	D_max_	≤ 115%	Hard
	D_max_	≤ 110%	Soft
	V_50.4Gy_	≥ 95%	Soft
bladder	V_50Gy_	< 50%	Hard
	V_40Gy_	< 60%	Soft
rectum	V_50Gy_	< 50%	Hard
	V_40Gy_	< 60%	Soft
bowel bag	V_45Gy_	< 195 cc	Hard
femoral head (L, R)	V_50Gy_	< 5%	Hard
kidney (L, R)	D_mean_	< 15 Gy	Hard
kidney (L, R)	D_mean_	< 10 Gy	Soft

L, Left; R, Right

#### M-TOMO planning

The clinical M-TOMO plans for the 30 patients were created using the Precision TPS with the TOMO Radixact Linac (Accuray Inc., Sunnyvale, CA, USA). These plans utilized the dynamic jaw mode with a jaw width set to 2.51 cm. Three plans used a pitch of 0.287, while the remaining 27 plans had a pitch of 0.43. The average Modulation Factor (MF) was 1.99 ± 0.41. Helical mode was employed, and dose calculation was performed using the collapsed cone algorithm, with the final dose calculation was performed in high-resolution mode. The radiotherapy plans for these 30 patients were randomly assigned to 14 dosimetrists, each employing potentially different manual optimization strategies. All plans were approved by oncologists for clinical treatment.

#### A-TOMO planning

##### Creation of auxiliary structures

**Fig. 1:**
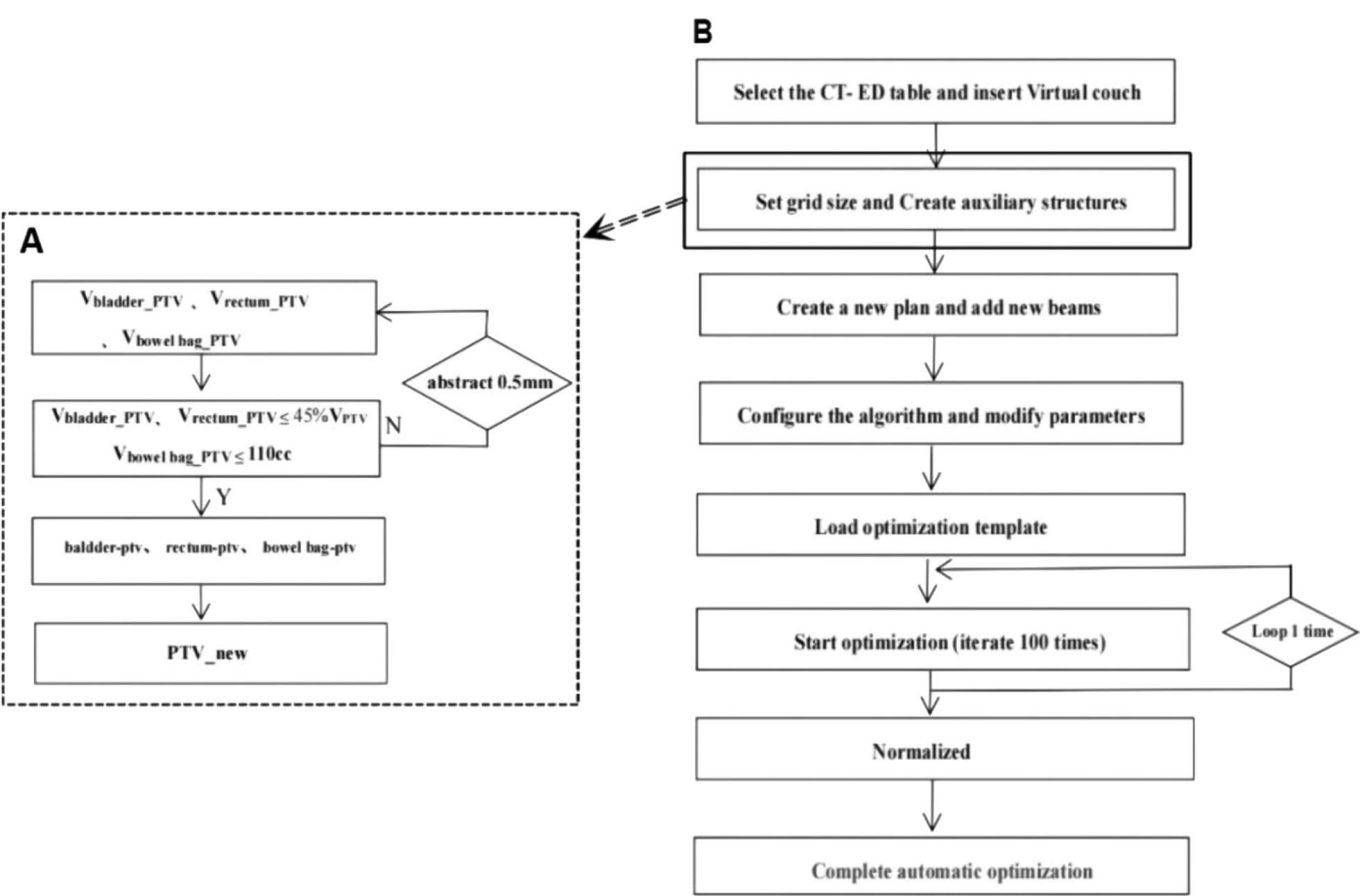
The workflow of the script-based automated planning method. **A** Flowchart for creating auxiliary structures. **B** Flowchart for the execution of the automated planning script. *Note*: V_bladder_PTV_, V_rectum_PTV_, and V_bowel bag_PTV_ represented the volumes of intersection between the bladder, rectum, and bowel bag with the PTV, respectively. The 'abstract 0.5mm' indicated that with each cycle, the PTV was contracted inward by 0.5 mm

**Fig. 2:**
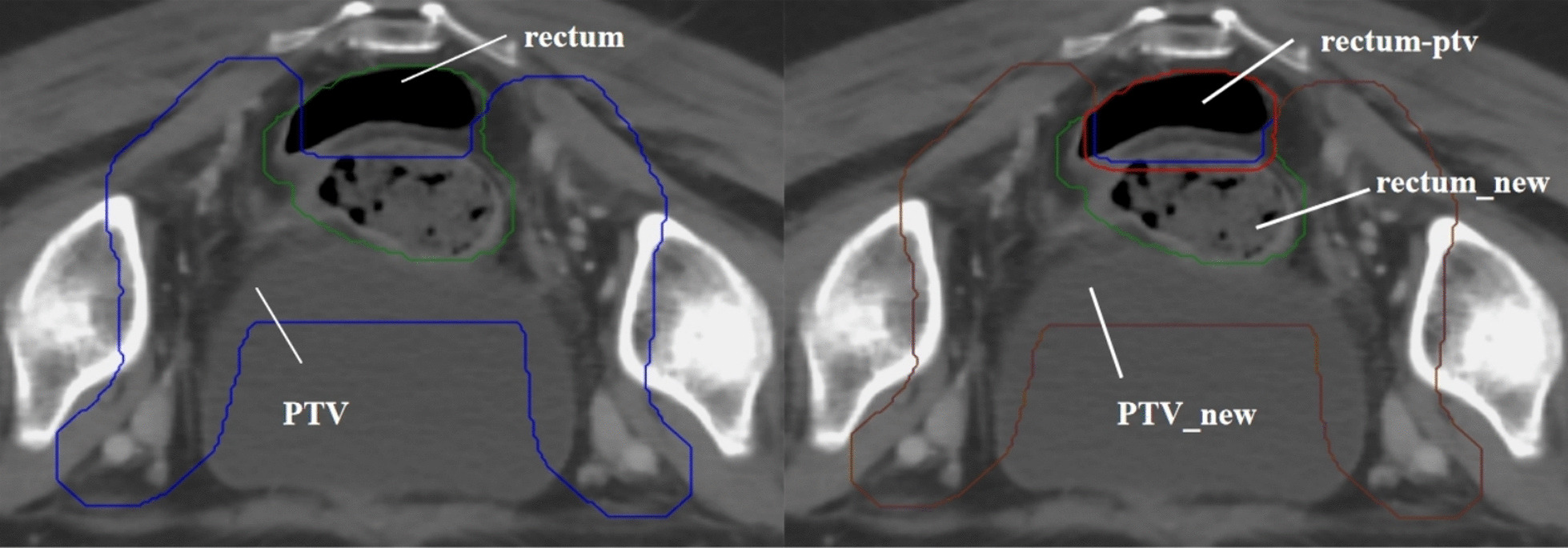
Examples of the auxiliary structure creation. Left: The green area represented the rectum, and the blue area represented the PTV. Right: The red area represented rectum-ptv, the brown area represented PTV_new, and the remaining green part represented rectum___new. Equations: rectum − rectum_new = rectum-ptv; PTV − (rectum-ptv) = PTV_new. *Note*: rectum_new represented the volume of intersection between the rectum and the PTV after it has been contracted inward

The method for creating auxiliary structures is detailed in Table 2. To facilitate meeting clinical dose requirements and to concentrate PTV dose deficits in areas overlapping with OARs as much as possible, we processed these overlapping sections as described in Fig. 1A. We ensured that the overlapping volumes of the rectum and bladder in the PTV were less than or equal to 45% of their total volume, and the overlapping volume of the bowel bag in the PTV was less than or equal to 110 cc, based on clinical requirements and experience. For example, the auxiliary structures PTV_new and rectum-ptv, created for preprocessing the overlapping section between the rectum and PTV, are shown in Fig. 2. To address potential dose control challenges caused by larger bowel volumes, we specifically created an auxiliary structure named "bag" to aid optimization. We set optimization goals based on each patient's bowel volume, calculated as (195/V_bag_)%, and took the integer part of the value.

**Table 2:** The creation method of auxiliary structures

Auxiliary structures	Creation method
ring0.5	Create ring of PTV with a 5 mm margin
ring1	Create ring of PTV between the 5 mm and 1cm margin
ring2	Create ring of PTV between the 1cm to 2 cm margin
ring3	Create ring of PTV between the 2 cm to 3 cm margin
nt	Subtract PTV and PTV rings from the body
PTV_new	Subtract the partial volume of the OARs from PTV
PTV_new-3mm	Create contraction of PTV_new with a 3 mm margin
bladder-ptv	Subtract PTV_new from bladder
rectum-ptv	Subtract PTV_new from rectum
bowel bag-ptv	Subtract PTV_new from bowel bag
bag	Create the overlap between bowel bag and PTV expanded outward by 2 cm
bowel bag_PTV	Create the overlap between bowel bag and PTV

##### Formulation of A-TOMO planning

The A-TOMO planning script was developed using the scripting function in RayStation 10B (RaySearch Labs, Sweden) TPS, within a Python (3.6) environment. This script utilized the standard model of the Radixact Linac for dose optimization and calculation. After verifying the contours and region of interest (ROI) names, the CT images were imported into the RayStation system, initiating the automated planning script. The workflow of the A-TOMO planning script is shown in Fig. 1B. Firstly, the script automatically selected the CT electron density conversion table and set the outline (body) type to “External”. A dose grid size of 0.3 × 0.3 × 0.3 cm was established. A virtual couch was inserted, and auxiliary structures were created. The script then automatically created a new plan and added beams, adjusting machine parameters, including setting the delivery time factor (DTF) to 1.7, the optimization stopping tolerance to 0, and the maximum number of iterations to 100. The system automatically loaded the optimization objective template, as listed in Table 3. The entire optimization process consisted of two rounds, each with 100 iterations of optimization. Ultimately, the plan was normalized so that the prescription dose covered 95% of the PTV volume, completing the optimization process. If the final normalized doses to OARs exceeded dose constraints, manual normalization was made by the dosimetrists as needed. Moreover, the A-TOMO planning script defaulted to a pitch of 0.43 and a jaw width of 2.51 cm, employing dynamic jaw mode and the collapsed cone dose calculation algorithm. The average MF for these plans was 1.83 ± 0.17.

**Table 3:** The optimization objective template

ROI	Type	Target (Gy)	Volume	Weight	EUD
PTV_new	Max Dose	52.4		1 × 10^9^	
PTV_new	Min DVH	51	97%	5 × 10^9^	
PTV_new	Min DVH	50.4	98%	5 × 10^9^	
PTV	Max EUD	52		1.5 × 10^9^	A = 150
PTV_new-3mm	Min Dose	51		8 × 10^9^	
ring0.5	Max Dose	50.4		3 × 10^8^	
ring1	Max Dose	45		3 × 10^8^	
ring2	Max Dose	38		3 × 10^8^	
ring3	Max Dose	28		3 × 10^8^	
nt	Max Dose	20		3 × 10^8^	
ring1	Max EUD	0		0.01	A = 1
ring2	Max EUD	0		0.01	A = 1
ring3	Max EUD	0		0.01	A = 1
nt	Max EUD	0		0.3	A = 1
bag	Max DVH	42	(195/V_bag_)%	3 × 10^9^	
bladder	Max DVH	38	60%	2 × 10^9^	
rectum	Max DVH	38	60%	2 × 10^9^	
bowel bag_PTV	Min Dose	40		2 × 10^9^	
bladder-ptv	Max EUD	0		1	A = 1
rectum-ptv	Max EUD	0		0.8	A = 1
bowel bag-ptv	Max EUD	0		5	A = 1
femoral head_L	Max EUD	0		0.5	A = 1
femoral head_R	Max EUD	0		0.5	A = 1
kidney_L	Max EUD	0		1	A = 1
kidney_R	Max EUD	0		1	A = 1

*P*^+^ indicated a comparison between A-TOMO and M-TOMO

*P*^−^ indicated a comparison between A-TOMO and A-VMAT

##### A-VMAT planning

The A-VMAT planning script was developed in the RayStation 10B TPS and Python (3.6) environment, employing an optimization strategy similar to that used for the A-TOMO plan. The automated plans were optimized based on a standard model for the TrueBeam Linac (Varian Medical Systems, Palo Alto, CA, USA) with 6 MV and a 120-leaf millennium multi-leaf collimator (MLC). In the A-VMAT planning script, two beams with gantry angles from 181° to 179° were automatically created, irradiating in both clockwise and counterclockwise directions. Moreover, the collimator angles were set to 355°. The maximum values for the jaws X1, X2, Y1, Y2 were set to 1, 14, 20, 20, and 14, 1, 20, 20 (cm), respectively, with jaw tracking mode, and the gantry spacing set at 2°. During optimization, the stopping tolerance was set to 0, employing the collapsed cone dose calculation algorithm with a calculation grid size of 0.3 × 0.3 × 0.3 cm. The maximum number of iterations was 100, with the iterations before conversion set at 40. The optimization process included two rounds, each consisting of 100 iterations. The system defaulted to optimizing segment shapes and segment MU.

### Evaluation

To evaluate the differences between radiotherapy plans, we conducted comparisons from two perspectives. Firstly, we compared A-TOMO plans with M-TOMO plans; secondly, we compared A-TOMO plans with A-VMAT plans. These comparisons primarily focused on the dosimetric indexes for PTV and OARs. For the assessment of OARs, our analysis concentrated on the dosimetric indexes for the bladder, rectum, and bowel bag (D_0.03cc_, V_50Gy_%, V_40Gy_%, D_mean_), femoral head_L, femoral head_R (V_50Gy_%, D_mean_) and kidney_L, kidney_R (D_mean_). For the PTV, we used D_max_, conformity index (CI), and homogeneity index (HI) for evaluation. Additionally, we assessed the dose drop-off outside the target volume using gradient index (GI_x_) for thresholds of 40 Gy, 30 Gy, 20 Gy, and 10 Gy. The CI, HI, GI_x_ were defined in the formulas below:

$${\text{HI}} = \frac{{{\text{D}}_{2{{\% }}} - {\text{D}}_{98{{\% }}}}}{{{\text{D}}_{50{{\% }}}}}$$

The D_2%_, D_98%_, and D_50%_ referred to the doses received by 2%, 98%, and 50% of the PTV volume in the DVH, respectively. A lower HI indicated better uniformity of the dose distribution within the PTV.

$${\text{CI}} = \frac{{{\text{TV}}_{{{\text{PTV}}}} \times {\text{ TV}}_{{{\text{PTV}}}} }}{{{\text{V}}_{{{\text{PTV}}}} {\text{ }} \times {\text{ V}}_{{{\text{TVP}}}} }}$$

The TV_PTV_ represented the volume within the PTV encompassed by the prescription dose line, and the V_PTV_ was the volume of the PTV, and the V_TVP_ was the volume encompassed by the prescription dose line. The closer the CI value was to 1, the better the conformity.

$${\text{GI}}_{{\text{x}}} = \frac{{{\text{V}}_{{{\text{x}}}} }}{{{\text{V}}_{{{\text{TVP}}}} {\text{ }} }}$$

The V_x_ referred to the volume encompassed by the x Gy dose line, while the V_TVP_ represented the volume encompassed by the prescription dose line. A smaller GI_x_ value indicated a faster dose drop-off outside the target volume.

Furthermore, we conducted a statistical analysis on the treatment delivery time as directly displayed by the TPS. To comprehensively evaluate work efficiency, we also assessed the time required to execute the automated treatment planning scripts.

### Statistical analysis

Before conducting the statistical analysis, we renormalized the A-TOMO and A-VMAT radiotherapy plans to ensure they matched the prescription dose coverage of the PTV as the M-TOMO plans. The data were found not to follow a normal distribution; hence, we employed the two-sided Wilcoxon signed-rank test as our statistical method, using the Wilcoxon function from the scipy.stats library in Python. In this analysis, a P-value less than 0.05 was considered statistically significant. To clearly present and compare the outcomes of different treatment plans, we recorded the mean values and standard deviations of each dataset.

## Results

### A-TOMO Plan versus M-TOMO plan comparison

**Fig. 3:**
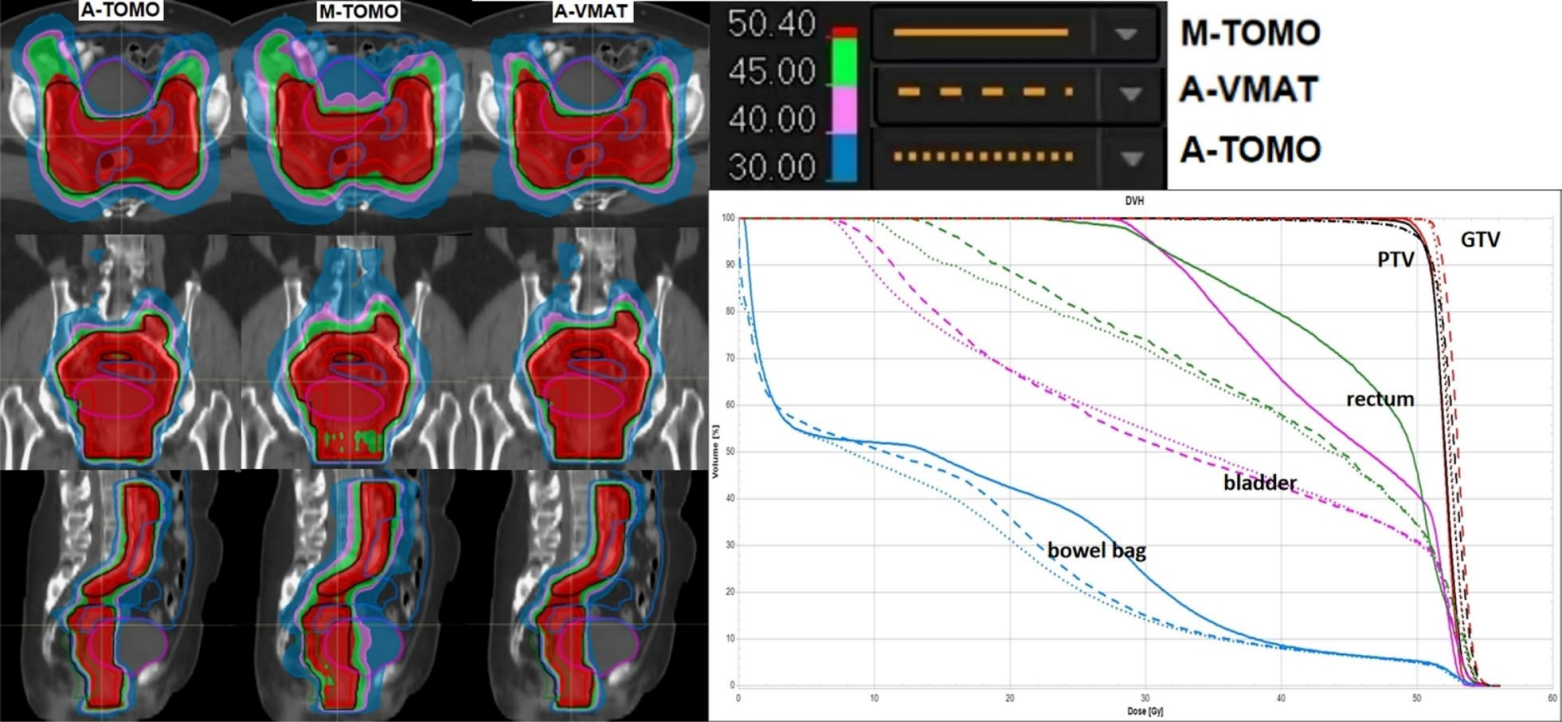
Dose Distributions and DVH of Three Plans of a case

In comparing the A-TOMO plans and M-TOMO plans for the 30 patients, it was found that for 5 patients, neither the manual nor the automated plans could achieve prescription dose coverage of 95% PTV, while meeting the dose constraints for OARs. The dosimetric comparison results are shown in Table 4. Although both plans demonstrated similar performance in terms of PTV D_max_ and HI (*P*^+^ = 0.92, 0.11), A-TOMO plans significantly surpassed M-TOMO plans in PTV CI (*P*^+^ < 0.01) and achieved greater reductions in OAR doses. Specifically, for the D_0.03cc_ for the bladder, rectum, and bowel bag, and the V_45Gy_ for the bowel bag, the two plans were comparable (*P*^+^ = 0.12, 0.57, 0.52 > 0.05; *P*^+^ = 0.11). However, for the V_50Gy_%, V_40Gy_%, and D_mean_ for the bladder and rectum, as well as the D_mean_ for the bowel bag, kidney_L, and kidney_R, A-TOMO plans were significantly lower than those in M-TOMO plans (*P*^+^ < 0.05). The V_50Gy_% for femoral head_L and femoral head_R in both plans were nearly identical, close to 0. Additionally, the GI_40Gy_, GI_30Gy_, and GI_20Gy_ values were significantly lower in A-TOMO plans than in M-TOMO plans (*P*^+^ < 0.01), indicating a faster dose drop-off outside the target volume in A-TOMO plans, thereby exposing normal tissues to lower radiation doses. The mean values ± standard deviations of the dose metrics also indicated better consistency in A-TOMO plans. Figure 3 presents a comparison of representative dose distribution and DVH for key ROIs for a case.

**Table 4:** Comparison of dosimetric indexes for three types of plans

ROI	Parameter	A-TOMO	M-TOMO	A-VMAT	*P*^+^	*P*^−^
PTV	D_max_ (Gy)	55.53 ± 0.63	55.55 ± 1.79	55.93 ± 0.55	0.92	< 0.01
	D_98%_ (Gy)	48.08 ± 1.90	48.4 ± 2.12	48.24 ± 1.90	< 0.01	0.45
	D_2%_ (Gy)	54.33 ± 0.50	54.19 ± 1.61	54.59 ± 0.62	0.49	< 0.01
	CI	0.91 ± 0.03	0.88 ± 0.06	0.90 ± 0.03	< 0.01	0.01
	HI	0.12 ± 0.04	0.11 ± 0.06	0.12 ± 0.04	0.11	0.06
bladder	D_0.03cc_ (Gy)	55.19 ± 0.60	54.73 ± 1.66	55.51 ± 0.74	0.12	< 0.01
	V_50Gy_ (%)	33.36 ± 7.32	37.54 ± 7.94	33.9 ± 7.67	< 0.01	< 0.01
	V_40Gy_ (%)	48.23 ± 8.59	63.61 ± 10.34	48.34 ± 8.41	< 0.01	0.16
	D_mean_ (Gy)	35.02 ± 4.22	42.69 ± 4.31	35.35 ± 4.10	< 0.01	0.01
rectum	D_0.03cc_ (Gy)	55.28 ± 0.57	55.11 ± 1.70	55.33 ± 0.75	0.57	0.20
	V_50Gy_ (%)	35.50 ± 5.08	40.55 ± 9.53	36.43 ± 5.52	0.03	< 0.01
	V_40Gy_ (%)	53.28 ± 6.38	68.54 ± 11.35	54.76 ± 5.75	< 0.01	< 0.01
	D_mean_ (Gy)	36.82 ± 2.40	46.50 ± 10.69	37.86 ± 2.10	< 0.01	< 0.01
bowel bag	D_0.03cc_ (Gy)	55.06 ± 0.54	54.90 ± 1.99	55.42 ± 0.73	0.52	< 0.01
	V_45Gy_ (cc)	141.83 ± 47.03	146.39 ± 42.2	141.76 ± 48.22	0.11	0.38
	D_mean_ (Gy)	10.94 ± 3.79	12.88 ± 4.72	11.22 ± 4.00	< 0.01	< 0.01
femoral head_L	V_50Gy_ (%)	0.01 ± 0.02	0.67 ± 2.37	0.00 ± 0.00	0.89	0.29
	D_mean_ (Gy)	15.98 ± 1.73	25.21 ± 6.16	17.81 ± 1.73	< 0.01	< 0.01
femoral head_R	V_50Gy_ (%)	0.00 ± 0.01	0.08 ± 0.23	0.00 ± 0.01	0.04	0.04
	D_mean_ (Gy)	15.51 ± 1.82	25.35 ± 5.73	16.92 ± 2.47	< 0.01	< 0.01
kidney_L	D_mean_ (Gy)	0.58 ± 2.83	1.04 ± 3.92	0.6 ± 3.55	< 0.01	0.14
kidney_R	D_mean_ (Gy)	0.65 ± 3.48	1.13 ± 4.24	0.63 ± 3.70	< 0.01	0.39
GI	GI_40Gy_	1.55 ± 0.08	1.72 ± 0.13	1.53 ± 0.10	< 0.01	< 0.01
	GI_30Gy_	2.30 ± 0.13	2.90 ± 0.26	2.28 ± 0.15	< 0.01	< 0.01
	GI_20Gy_	4.64 ± 0.29	5.42 ± 0.43	4.71 ± 0.33	< 0.01	< 0.01
	GI_10Gy_	9.54 ± 1.33	9.55 ± 1.47	9.30 ± 1.23	0.61	< 0.01

### A-TOMO plan versus A-VMAT plan comparison

The dosimetric comparison results between A-TOMO and A-VMAT plans for the 30 patients are shown in Table 4. The A-VMAT plan showed a slight advantage over the A-TOMO plan in terms of the GI, but it presented slightly higher values for the PTV (D_max_, D_2%_), bladder (D_0.03cc_, V_50Gy_%, D_mean_), rectum (V_50Gy_%, V_40Gy_%, D_mean_), bowel bag (D_0.03cc_, D_mean_), femoral head_L (D_mean_), and femoral head_R (V_50Gy_%, D_mean_). Although these differences were statistically significant (*P*^−^ < 0.05), the actual numerical differences were within 1 Gy and 1%. This suggests that the A-TOMO plan was comparable in overall plan quality to the A-VMAT plan, and may even be superior. Representative dose distributions and DVH for key ROIs of the case are displayed in Fig. 3.

Regarding the workflow efficiency, the execution time for the A-TOMO planning script was approximately only 20 min, whereas that for the A-VMAT planning script took slightly longer, about 30 min. In terms of treatment delivery efficiency, as reported by the TPS and shown in Table 5, the dose delivery time for A-TOMO plans was slightly increased compared to M-TOMO plans. In contrast, A-VMAT plans demonstrated superior dose delivery efficiency, requiring less than 3 min, which was significantly less than the time required for TOMO plans.

**Table 5:** Treatment delivery parameters

Beam delivery parameters	A-TOMO	M-TOMO	A-VMAT	*P*^+^	*P*^−^
Delivery time(min)	7.76 ± 0.95	5.99 ± 1.42	2.63 ± 0.04	< 0.01	< 0.01

*P*^+^ indicated a comparison between A-TOMO and M-TOMO;

*P*^−^ indicated a comparison between A-TOMO and A-VMAT

## Discussion

In this study, we successfully developed and validated the first automated Tomotherapy planning method for cervical cancer. This automated planning strategy not only significantly improved the quality of the plans but also enhanced work efficiency. The automated planning method we proposed could automatically generate personalized dose distributions based on the unique anatomical characteristics of different patients, offering greater generalization. The entire process required almost no manual intervention and was compatible with commercial TPS, which was easily applicable in clinical routine.

A fundamental principle of radiotherapy is ensuring adequate dose coverage to the target while minimizing the dose to OARs and normal tissue, thereby reducing unnecessary radiation-induced harm to the patient. Our study results demonstrated that A-TOMO plans significantly improved target dose conformity without sacrificing homogeneity, and reduced the dose to OARs and normal tissues compared to clinical M-TOMO plans. This was particularly true for critical indexes such as the V_50Gy_%, V_40Gy_% and D_mean_ for the rectum and bladder, as well as the D_mean_ for the bowel bag. Although no statistical significance was observed in the V_45Gy_ (cc) for the bowel bag between the two types of plans, the mean value for A-TOMO plans was 141.83 cc, lower than the 146.39 cc for the M-TOMO plans. Studies indicated that further reducing the dose to the small intestine and rectum could help decrease gastrointestinal toxicity [24–26].

The improvements in plan quality are influenced by the experience and effort of different dosimetrists and could also be affected by differences between TPS. Notably, differences exist between the Precision TPS and RayStation TPS, with Precision TPS lacking the equivalent uniform dose (EUD) function and allowing a maximum of only three optimization objectives for each ROI. Moreover, in the TOMO optimization process within Precision TPS, each voxel is assigned to only one ROI. Considering that the quality of M-TOMO plans could inevitably be influenced by subjective human factors, we compared A-TOMO plans with A-VMAT plans. The study results showed that the quality of these two types of plans was comparable, further affirming the plan quality of A-TOMO planning. A study by Panda et al. [27] suggested that VMAT and TOMO were equivalent in treating cervical cancer. A finding corroborated by the experimental data from Marnitz et al. [28], also aligned with our study results. These indicated that the A-TOMO planning method we developed further standardized TOMO planning, effectively showcasing the capabilities of TOMO technique.

**Fig. 4:**
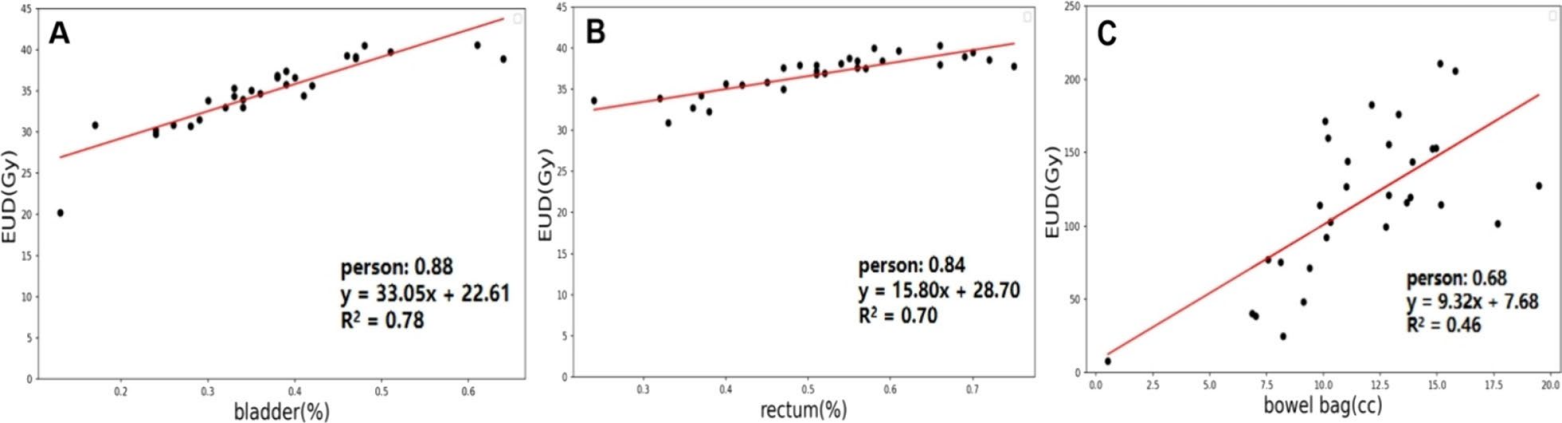
Regression Model of EUD (A = 1). **A** Logistic regression of the overlap rate between the bladder and PTV with the bladder's EUD; y = 33.05x + 22.61 was the fitted linear equation. **B** Logistic regression of the overlap rate between the rectum and PTV with the rectum's EUD; y = 15.80x + 28.70 was the fitted linear equation. **C** Logistic regression of the overlap volume between the bowel bag and PTV with the bowel bag's EUD; the linear correlation was not strong

Dose-volume parameters are simplified substitutes for potential biological effects and didn’t necessarily reflect the entire treatment region's dose distribution [29]. Combining dose-volume constraints with EUD could yield better dose distributions [30]. We set the EUD objective value to 0, as a previous study [31]. Additionally, studies showed that automated plans based on predicted EUD values were superior in quality to manual plans [32, 33]. We analyzed the relationship between the overlap of bladder, rectum, bowel bag and PTV with EUD (A = 1) in A-TOMO plans, as shown in Fig. 4. The Pearson correlation coefficients for bladder and rectum were 0.88 and 0.84, respectively, with R2 values of 0.78 and 0.70, indicating a strong linear relationship. It suggested that automated planning based on the predicted EUD method had research potential and was worth further exploration.

One of the significant features of the automated planning was its high efficiency, and our study results were consistent with this observation [34–36]. In our study, A-TOMO and A-VMAT planning only required about 20 min and 30 min to complete, respectively. However, as described by dosimetrists, the M-TOMO plans required repeated adjustments and could take several hours from start to submission. Our script set two consecutive rounds of iteration, each with 100 iterations, to seek the final solution. Although we had not yet delved into the possibility of achieving an optimal solution with fewer iterations, this exploration might further shorten the script execution time. In the future, incorporating contouring into the automated planning process could realize a more complete automated planning workflow, further saving time and improving efficiency.

Although the A-TOMO plan improved work efficiency, the treatment delivery time was longer compared to M-TOMO plans. This could be due to several reasons, including more stringent dose constraints and the DTF parameter in A-TOMO plans being set to 1.7, which made the execution time of each rotation approximately 20 s. A previous study indicated that increasing the DTF, while improving plan quality, also led to longer dose delivery times [37]. However, this did not increase the modulation of the A-TOMO plan. As shown in Table 6, the average MF value for A-TOMO was 1.83 ± 0.17, which is slightly lower than the average MF value of 1.99 ± 0.41 for M-TOMO plans, though the difference was not statistically significant. Additionally, compared to TOMO plans, VMAT plans had shorter dose delivery times, consistent with previous studies [38].

**Table 6:** Modulation factor

	A-TOMO	M-TOMO	*P*
Modulation factor	1.83 ± 0.17	1.99 ± 0.41	0.21

*P* indicated a comparison between A-TOMO and M-TOMO

Currently, the quality of our developed A-TOMO plan has been preliminarily validated in our treatment center. To further demonstrate its effectiveness and applicability, we plan to collaborate with multiple treatment centers for broader validation studies. This cross-center collaboration will provide a detailed assessment of our A-TOMO planning method, ensuring its stability and reliability in different settings. Through future multi-center collaboration, we aim to provide a comprehensive and precise validation platform for automated cervical cancer radiation therapy planning. Moreover, given the A-TOMO planning method's ability to generate high-quality and consistent plans, it has great potential to serve as a superior data source for training automated planning systems based on the atlas model. Compared to methods that use traditional manual planning as the training set, we anticipate not only improving the model's performance but also creating higher-quality plans.

## Conclusion

We have successfully developed an automated Tomotherapy planning method in RayStation TPS for external beam radiotherapy of cervical cancer. This method not only effectively improved the quality of the plans but also significantly enhanced work efficiency. Compared to M-TOMO plans, the A-TOMO plans achieved a higher level of plan quality while significantly reducing the dose to OARs. Moreover, A-TOMO plans demonstrated dose distributions similar to those of A-VMAT plans, further validating their quality and feasibility.

## Data Availability

The datasets used during the current study are available from the corresponding author on reasonable request.

## Abbreviations

TOMO: Tomotherapy
VMAT: Volumetric modulated arc therapy
M-TOMO: Manual TOMO
A-TOMO: Automated TOMO
A-VMAT: Automated VMAT
TPS: Treatment planning system
OARs: Organs at risk
PTV: Planning target volume
RTOG: Radiotherapy Oncology Group
CTV: Clinical target volume
NCCN: National Comprehensive Cancer Network
QUANTEC: Quantitative Analysis of Normal Tissue Effects in the Clinic
DVH: Dose volume histogram
ROI: Region of interest
DTF: Delivery time factor
MLC: Multi-leaf collimator
CI: Conformity index
HI: Homogeneity index
GI: Gradient index
EUD: Equivalent uniform dose
MF: Modulation factor
